# A new orthopteran-parasitizing horsehair worm, *Acutogordius taiwanensis* sp. n., with a redescription of *Chordodes formosanus* and novel host records from Taiwan (Nematomorpha, Gordiida)

**DOI:** 10.3897/zookeys.683.12673

**Published:** 2017-07-06

**Authors:** Ming-Chung Chiu, Chin-Gi Huang, Wen-Jer Wu, Shiuh-Feng Shiao

**Affiliations:** 1 Department of Entomology, National Taiwan University, Taipei 106, Taiwan; 2 Department of Earth and Life Science, University of Taipei, Taipei 100, Taiwan

**Keywords:** *Acutogordius taiwanensis*, *Chordodes formosanus*, immature stage, new species, novel host

## Abstract

A description of a new species of horsehair worm, *Acutogordius taiwanensis* **sp. n.**, a redescription of *Chordodes formosanus*, and novel host records for the latter are provided. *Acutogordius taiwanensis* **sp. n.** is morphologically similar to *A. protectus* with moderately flat areoles on its tail tips, but is distinguishable by small mid-body ornamentations. Despite the distinct differences in the post-cloacal crescents between 14 male samples, their conspecific status, along with that of nine female samples, was upheld by a phylogenetic comparison of partial cytochrome oxidase subunit I (COI) sequences. *Chordodes formosanus* is another common horsehair worm species in Taiwan, which was previously believed to specifically parasitize *Hierodula* mantids. However, in this study, five *C. formosanus* were observed emerging from an *Acromantis* mantid, and two long-horned grasshopper hosts (*Leptoteratura* sp. and *Holochlora japonica*). These five worms showed high degrees of similarity in COI sequences and morphology, but one of these individuals bore abnormal crowned areoles, which has never been observed in *C. formosanus*, and may be attributed to the incomplete development of this particular individual.

## Introduction

Horsehair worms (phylum Nematomorpha) are aquatic parasites whose life cycle typically contains a free-living, aquatic phase, including mating and early larval development, and two parasitic stages, including an aquatic paratenic host stage and a terrestrial definitive host stage (Schmidt-Rhaesa 2014, Bolek et al. 2015). Over 350 freshwater species have been described worldwide (Bolek et al. 2015), but only one, *Chordodes formosanus* Chiu, 2011, is known in Taiwan (Chiu et al. 2011). *Acutogordius taiwanensis* sp. n. is another commonly encountered species, which is usually sympatric with *C. formosanus* at low altitudes in Taiwan. In the aquatic paratenic host stage, cysts of *Acutogordius* and *Chordodes* have been found sympatrically in aquatic chironomids (Chiu et al. 2016), whereas the adults generally parasitize various terrestrial hosts.

The definitive hosts of *C. formosanus* are *Hierodula* mantids (Chiu et al. 2011), whereas adults of *Acutogordius taiwanensis* sp. n. in Taiwan generally emerge from orthopteran insects. *Acutogordius* is a small genus that consists of ten described species (de Miralles and de Villalobos 1998, Schmidt-Rhaesa and Geraci 2006, Schmidt-Rhaesa and Schwarz 2016) in the family Gordiidae (Poinar 2008). Only two genera in the Gordiidae, *Acutogordius*, and *Gordius*, are characterized by a post-cloacal crescent located at the base of the two tail lobes, but are distinguishable by the distinctly pointed tips on the *Acutogordius* male’s tail lobes (Schmidt-Rhaesa 2002). Classification of *Acutogordius* species is primarily based on the characters of the males’ tails. However, interspecific variation has not been well defined, and distinction between the species is still not clear (Schmidt-Rhaesa and Geraci 2006).

In the present study, the conspecific status of 23 *Acutogordius* samples is established according to minor differences observed in the sequences of their partial mitochondrial DNA cytochrome oxidase subunit I (mtDNA-COI) genes, collected from eleven species of orthopteran hosts, and the morphological differences among these samples is determined as intraspecific variation. Furthermore, the morphology of the immature stages of this species was also described. Five horsehair worm samples that had emerged from *Acromantis* mantids and two long-horned grasshopper species, i.e., *Leptoteratura* sp. and *Holochlora japonica*, were identified as *C. formosanus* based on morphological and molecular evidence, and thus added these insect species as novel definitive hosts of *C. formosanus*.

## Materials and methods

The morphologies and DNA sequences of 29 adult horsehair worms (24 *Acutogordius* and 5 *Chordodes*) were examined. Two pairs of *Acutogordius* were reared in the laboratory for two weeks to breed and lay eggs, and the morphology of the resulting larvae was examined using a light microscope (Olympus BH-2, PM-10AD, Tokyo, Japan). Specimens (partial bodies with their hosts) were preserved at the Department of Entomology, National Taiwan University, Taipei; National Museum of Natural Science, Taichung, Taiwan; and Lake Biwa Museum, Shiga, Japan. Specimen examination followed our previously published methods described in Chiu et al. (2011).

### Collection and preservation of horsehair worms

Insect hosts infected with horsehair worms were hand-collected from a riparian environment. To determine if a host was infected with worms, its posterior was examined, and then the worms were collected by immersing the host in water or by host dissection. Except for the two pairs of *Acutogordius* that were kept for breeding, all other horsehair worms were killed with hot water (>80°C), fixed in a 75% alcohol solution with their hosts for few days, and preserved in a 95% alcohol solution. The collection and host data are given in Tables 1 and 2, respectively.

Breeding *Acutogordius* pairs were reared in 800 mL aerated tap water and maintained at room temperature (~28°C). After mating, the males were removed and fixed and preserved in 75% followed by 95% alcohol solutions. After the mated females laid eggs for one month, they were also removed and fixed and preserved in 75% followed by 95% alcohol solutions. Egg strings were detected at approximately three days, and hatched larvae were detected at approximately 2–3 weeks after egg laying. Live larvae were observed under a light microscope.

Snails (*Physa* sp.) infected by horsehair worms were collected with nine other non-infected snails from a small pond in Wufengqi Waterfalls, Jiaushi Township, Yilan County, Taiwan, where free-living adult horsehair worms have been seen. Live snails were maintained together in 2000 mL aerated tap water and were dissected after five days.

### Morphological examination

*Adult specimens.* Fragments (~0.5 cm in length) of the anterior end, mid-body, and posterior end of the preserved adult horsehair worm samples were first examined under a stereomicroscope (Leica S8 APO, Leica, Wetzlar, Germany). The fragments were dehydrated with a series of ethanol and acetone solutions (95% and 100% ethanol (twice) and ethanol/acetone mixtures of 2:1, 1:1, 1:2, 0:1) and critical-point-dried and gold-sputter-coated before being examined under a scanning electronic microscope (SEM) (JEOL JSM-5600, Tokyo, Japan) at a magnification of 100–15,000×.

*Immature stages.* Eggs and newly hatched larvae were examined and photographed alive on microslides using a light microscope (Olympus BH-2, PM-10AD, Olympus, Tokyo, Japan) at magnifications of 200× and 400×. Larvae, for examination under SEM, were first killed, fixed by 75% alcohol solution, and collected in a paper envelope soaked in 75% alcohol solution. The protocol of dehydration, critical point drying, and gold sputter coating followed that applied in the examination of the adult fragments and they were examined at a magnification of 500–9,000×. To examine the cysts inside the snail hosts, the snail shells were removed, the soft tissue flattened by two glass slides, and the slides were examined under a light microscope at 200× magnification.

The measurements of each characteristic were performed using the segmented line function in ImageJ 1.47 (Abràmoff et al. 2004), and calibrated spatially to the scale included in each picture. The terminology for larval stages used in this study primarily followed that of Hanelt and Janovy (2002) and Szmygiel et al. (2014).

### Phylogenetic analysis

Genomic DNA from adult horsehair worms was extracted using an ALS Tissue Genomic DNA Extraction Kit (Pharmigene, Kaohsiung, Taiwan). A set of universal primers (LCO1490 and HC02198) (Folmer et al. 1994) were applied to amplify and sequence the partial COI sequence. Fourteen *Acutogordius* samples with COI sequences that could not be well amplified with the universal primers were prepared for use with a newly designed primer set (AcCOiF: TGAGCTGCCTTTTTAG, AcCOiR: TGTATTAATGTTTCGGTC). The PCR for both primer sets was initiated at 95°C for 5 min, and amplification was conducted for 40 cycles of 95°C for 1 min, 50°C for 1 min, and 72°C for 1 min, with a final extension at 72°C for 10 min.

Pairwise genetic distances and phylogenic tree reconstruction using the neighbor-joining (NJ) method, which is based on Kimura’s 2-parameter model, were used to verify conspecific status of the horsehair worm samples. COI sequences (450 high-quality nucleotide base pairs) were first aligned using CLUSTALX 2.0.10 (Thompson et al. 1997), and the analysis was conducted with MEGA 6.0 (Tamura et al. 2013). COI sequences for *Gordius balticus*, *G. attoni*, and G. cf. robustus. (Hanelt et al. 2015, GenBank nos. KM382320, KM382318, KM382277), *C. formosanus* and *C. japonensis* (Chiu et al. 2011, GenBank nos HM044105, HM044124, JF808206), and *Paragordius* sp. (GenBank no. AY428843) were also used in the comparison, and 5,000 bootstrap replicates were used to determine branch supports for the NJ tree.

## Taxonomy

### Acutogordius taiwanensis sp. n.

Taxon classificationAnimaliaNematomorphaGordiida

http://zoobank.org/59379D36-D879-4F8D-BB78-C47C88F818DB

#### Type locality.

Wufengqi Waterfalls (24°49′55.62′′N, 121°44′50.10′′E), Jiaushi Township, Yilan County, Taiwan (holotype and allotype). Paratypes were collected from Sindian, New Taipei City, and the Fushan Botanical Garden, Yilan County. See Table 1 for detailed information.

#### Type material.

Partial bodies of the holotype and allotype were deposited with their hosts at the National Museum of Natural Science. Paratypes were deposited at the Department of Entomology, National Taiwan University, Taipei; the National Museum of Natural Science, Taichung, Taiwan; and Lake Biwa Museum, Shiga, Japan. See Table 1 for detailed information.

**Table 1.** *Acutogordius taiwanensis* sp. n. and *Chordodes formosanus* specimen information.

Horsehair worm									
Species	Collection date	GenBank no.	Locality	Longitude and latitude	Collector	Depository	Sex	Length (mm)	Host code
*A. taiwanensis*	16-XI-2014	KX591947	Xindian, New Taipei, Taiwan	24°50'47.70"N 121°32'41.20"E	Shipher Wu	NTU	F	283	HAc23302
*A. taiwanensis*	2-VIII-2009	KX591922	Jiaushi, Yilian, Taiwan	24°49'55.62"N, 121°44'50.12"E	Ming-Chung Chiu	NTU	M	334	HAc26201
*A. taiwanensis*	29-VII-2009	KX591948	Fushan botanical garden, Yilian, Taiwan	-	Ming-Chung Chiu	NTU	M	278	HAC26401
*A. taiwanensis*	10-VII-2011	KX591926	Jiaushi, Yilian, Taiwan	24°49'55.62"N, 121°44'50.12"E	Ming-Chung Chiu	NTU	M	312	HAc26206
*A. taiwanensis*	5-VII-2011	**KX591927^1^**	Jiaushi, Yilian, Taiwan	24°49'55.62"N, 121°44'50.12"E	Ming-Chung Chiu	NMNS	M	410	HAc26207
*A. taiwanensis*	5-VII-2011	KX591928	Jiaushi, Yilian, Taiwan	24°49'55.62"N, 121°44'50.12"E	Ming-Chung Chiu	NTU	M	428	HAc26208
*A. taiwanensis*	18-VIII-2011	KX591929	Jiaushi, Yilian, Taiwan	24°49'55.62"N, 121°44'50.12"E	Ming-Chung Chiu	NTU	F	360	HAc26209
*A. taiwanensis*	20-VII-2010	KX591930	Jiaushi, Yilian, Taiwan	24°49'55.62"N, 121°44'50.12"E	Ming-Chung Chiu	NTU	M	387	HAc26210
*A. taiwanensis*	24-IX-2011	KX591931	Jiaushi, Yilian, Taiwan	24°49'55.62"N, 121°44'50.12"E	Ming-Chung Chiu	LBM	M	262	HAc26211-12
*A. taiwanensis*	24-IX-2011	KX591932	Jiaushi, Yilian, Taiwan	24°49'55.62"N, 121°44'50.12"E	Ming-Chung Chiu	LBM	F	272	HAc26211-12
*A. taiwanensis*	5-VIII-2012	**KX591933^2^**	Jiaushi, Yilian, Taiwan	24°49'55.62"N, 121°44'50.12"E	Ming-Chung Chiu	NMNS	F	288	HAc26214
*A. taiwanensis*	21-VII-2012	KX591934	Jiaushi, Yilian, Taiwan	24°49'55.62"N, 121°44'50.12"E	Ming-Chung Chiu	NMNS	M	133	HAc26215
*A. taiwanensis*	21-XI-2012	KX591935	Jiaushi, Yilian, Taiwan	24°49'55.62"N, 121°44'50.12"E	Ming-Chung Chiu	NMNS	M	241	HAc26217
*A. taiwanensis*	31-VIII-2012	KX591937	Jiaushi, Yilian, Taiwan	24°49'55.62"N, 121°44'50.12"E	Ming-Chung Chiu	NMNS	M	222	HAc26219-20
*A. taiwanensis*	31-VIII-2012	KX591938	Jiaushi, Yilian, Taiwan	24°49'55.62"N, 121°44'50.12"E	Ming-Chung Chiu	NMNS	M	216	HAc26219-20
*A. taiwanensis*	31-VIII-2012	KX591939	Jiaushi, Yilian, Taiwan	24°49'55.62"N, 121°44'50.12"E	Ming-Chung Chiu	NMNS	F	322	HAc26221-21A
*A. taiwanensis*	31-VIII-2012	NA^3^	Jiaushi, Yilian, Taiwan	24°49'55.62"N, 121°44'50.12"E	Ming-Chung Chiu	NMNS	F	73	HAc26221-21A
*A. taiwanensis*	31-VIII-2012	KX591940	Jiaushi, Yilian, Taiwan	24°49'55.62"N, 121°44'50.12"E	Ming-Chung Chiu	NMNS	F	285	HAc26222
*A. taiwanensis*	26-VII-2014	KX591941	Jiaushi, Yilian, Taiwan	24°49'55.62"N, 121°44'50.12"E	Ming-Chung Chiu	LBM	M	369	HAc26223
*A. taiwanensis*	26-VI-2015	KX591942	Jiaushi, Yilian, Taiwan	24°49'55.62"N, 121°44'50.12"E	Ming-Chung Chiu	NTU	M	164	HAc26225-26
*A. taiwanensis*	26-VI-2015	KX591943	Jiaushi, Yilian, Taiwan	24°49'55.62"N, 121°44'50.12"E	Ming-Chung Chiu	NTU	F	166	HAc26225-26
*A. taiwanensis*	17-VII-2015	KX591944	Jiaushi, Yilian, Taiwan	24°49'55.62"N, 121°44'50.12"E	Ming-Chung Chiu	LBM	M	280	HAc26228
*A. taiwanensis*	17-VII-2015	KX591945	Jiaushi, Yilian, Taiwan	24°49'55.62"N, 121°44'50.12"E	Ming-Chung Chiu	LBM	F	432	HAc26231
*A. taiwanensis*	17-VII-2015	KX591946	Jiaushi, Yilian, Taiwan	24°49'55.62"N, 121°44'50.12"E	Ming-Chung Chiu	LBM	F	237	HAc26232
*C. formosanus*	11-II-2015	KX591949	Taipei Zoo, Taipei City, Taiwan	24°59'44.70"N, 121°34'49.49"E	Long-Chun Huang	NMNS	M	58	HCH11606-8
*C. formosanus*	11-II-2015	KX591950	Taipei Zoo, Taipei City, Taiwan	24°59'44.70"N, 121°34'49.49"E	Long-Chun Huang	NMNS	M	125	HCH11606-8
*C. formosanus*	11-II-2015	KX591951	Taipei Zoo, Taipei City, Taiwan	24°59'44.70"N, 121°34'49.49"E	Long-Chun Huang	NMNS	M	115	HCH11606-8
*C. formosanus*	4-III-2015	KX591952	Jiaushi, Yilian, Taiwan	24°49'55.62"N, 121°44'50.12"E	Ming-Chung Chiu	NTU	M	43	HCH26207
*C. formosanus*	10-XI-2015	KX591953	Jiaushi, Yilian, Taiwan	24°49'55.62"N, 121°44'50.12"E	Ming-Chung Chiu	NTU	M	204	HAc26216

LBM: Lake Biwa Museum; NMNS: National Museum of Natural Science; NTU: National Taiwan University

^1^ Holotype

^2^ Allotype

^3^ Female specimen with insufficient DNA for sequencing

#### Type hosts.

*Eugryllacris* sp., *Neanias magnus* Matsumura and Shiraki, 1908 (Orthoptera: Gryllacrididae), *Deflorita apicalis* (Shiraki, 1930), *Elimaea* sp., *Hexacentrus japonicus* Karny, 1907, *H. unicolor* Serville, 1831, *Isopsera* sp., *Mecopoda elongata* (Linnaeus, 1758), *Phaulula* sp., *Pyrgocorypha formosana* Matsumura and Shiraki, 1908, *Sinochlora longifissa* (Matsumura and Shiraki, 1908) (Orthoptera: Tettigoniidae). See Table 2 for detailed information.

**Table 2.** Mantid and grasshopper horsehair worm host information.

Host code (see Table 1)	Host species	Host sex	Host length (mm)
HAc23302	*Mecopoda elongata*	M	31.5
HAc26201	*Eugryllacris* sp.	M	27.5
HAC26401	*Neanias magnus*	F	20.5
HAc26206	*Neanias magnus*	M	21.9
HAc26207	*Eugryllacris* sp.	M	27.2
HAc26208	*Eugryllacris* sp.	M	25.9
HAc26209	*Hexacentrus japonicus*	F	29.4
HAc26210	*Sinochlora longifissa*	F	33.5
HAc26211-12	*Hexacentrus unicolor*	F	28.8
HAc26214	*Elimaea* sp.	F	27.1
HAc26215	*Deflorita apicalis*	M	22.3
HAc26217	*Pyrgocorypha formosana*	F	41.3
HAc26219-20	*Phaulula* sp.	F	23.1
HAc26221-21A	*Hexacentrus unicolor*	F	23.9
HAc26222	*Hexacentrus unicolor*	F	29.1
HAc26223	*Elimaea* sp.	F	27.2
HAc26225-26	*Neanias magnus*	M	17.9
HAc26228	*Hexacentrus unicolor*	M	28.4
HAc26231	*Eugryllacris* sp.	F	30.6
HAc26232	*Isopsera* sp.	M	24.1
HCH11606-8	*Acromantis japonica*	F	29.4
HCH26207	*Leptoteratura* sp.	F	9.6
HAc26216	*Holochlora japonica*	F	39.2

#### Etymology.

The specific name refers to the type locality, Taiwan.

#### Diagnosis.

*Acutogordius taiwanensis* sp. n. is morphologically similar to *A. protectus* Schmidt-Rhaesa and Geraci, 2006 with regards to the (1) distribution pattern of tiny bristles on the ventral posterior end, (2) moderately flat areoles (rounded in elevation) covering the tail tips, and (3) cone-shaped spines scattered on the base of the tail lobes of the male samples. However, it is distinct because of the small ornamentations on the mid-body.

**Figure 1.**
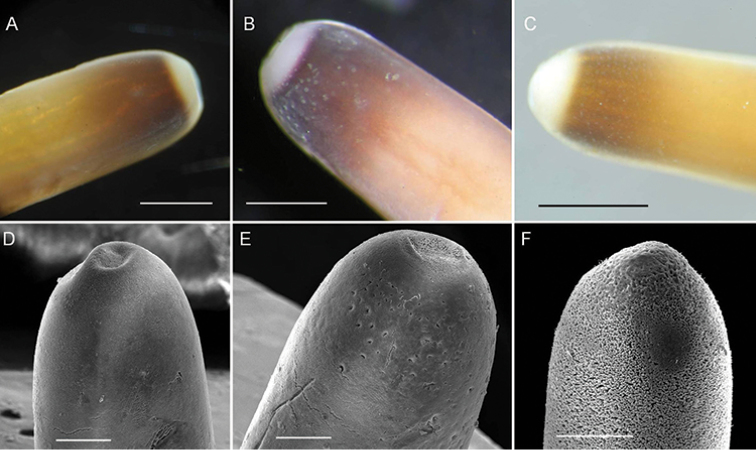
Anterior end of *Acutogordius taiwanensis* sp. n. **A–C** Images of the anterior end showing the (**A**) white cap and dark-brown collar and **B–C** white spots scattered on the brown collar **D–F** SEM images of the anterior end surface that is **D** smooth **E** smooth but wrinkled on the tip with holes scattered on the dark-brown collar, and **F** wrinkled **A–F** are images from the same individual, respectively. Scale bars 500 µm (**A–C**), and 200 μm (**D–F**).

**Figure 2.**
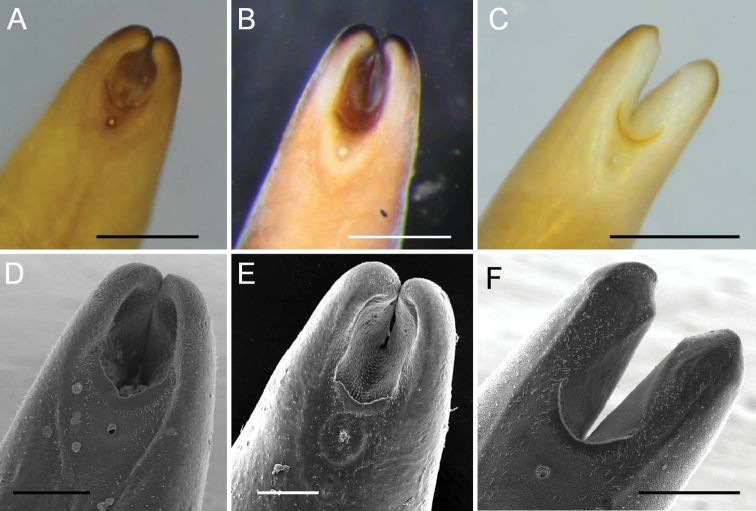
Posterior end of male *Acutogordius taiwanensis* sp. n. **A–C** Images of the posterior end with the postcloacal crescent extending **A, C** over or **B** anterior to the starting point of the tail lobe bifurcation **D–F** SEM images of the posterior end with a **D** angled **E** slightly curved, and **F** semicircular postcloacal crescent **A–F** are images from the same individual, respectively. Scale bars 500 µm (**A–C**), and 200 μm (**D–F**).

**Figure 3.**
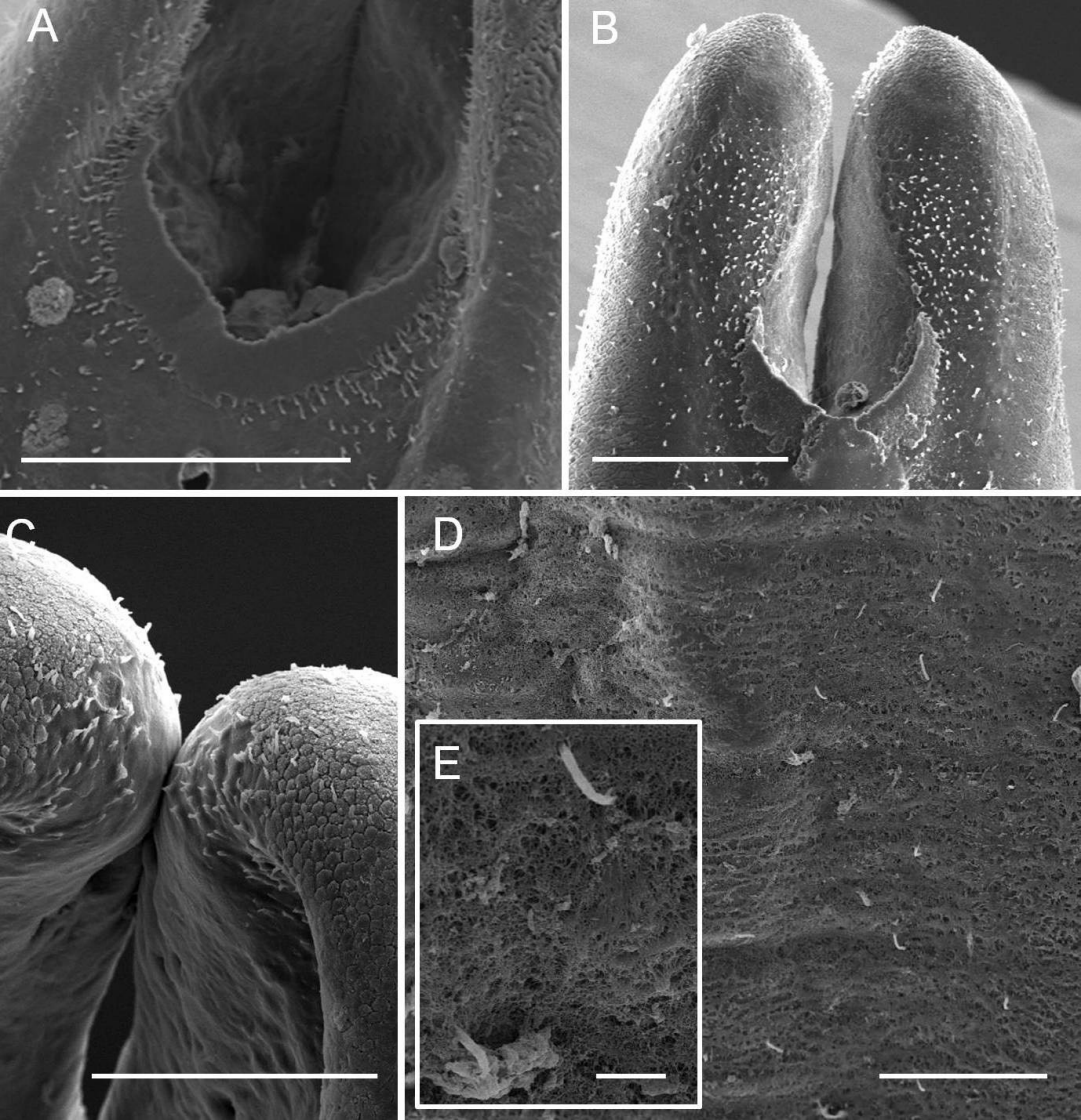
Detailed diagnostic characteristics of male *Acutogordius taiwanensis* sp. n. **A** Tiny bristles scattered anterior to postcloacal crescent **B** Tiny bristles scattered in concentrated groups on tail lobes **C** Lobe tips covered by moderately flat areoles with short spines amongst areoles **D–E** Short bristles scattered on the mid-body cuticle. Scale bars 200 µm (**A–B**), 100 µm (**C–D**), and 10 μm (**E**).

**Figure 4.**
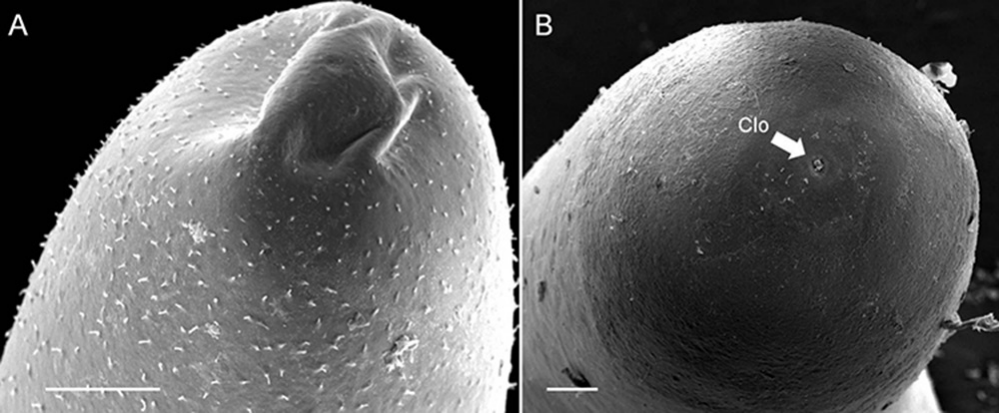
Female *Acutogordius taiwanensis* sp. n. **A** Anterior end **B** Posterior end. Clo, cloacal opening. Scale bars 100 µm (**A–B**).

**Figure 5.**
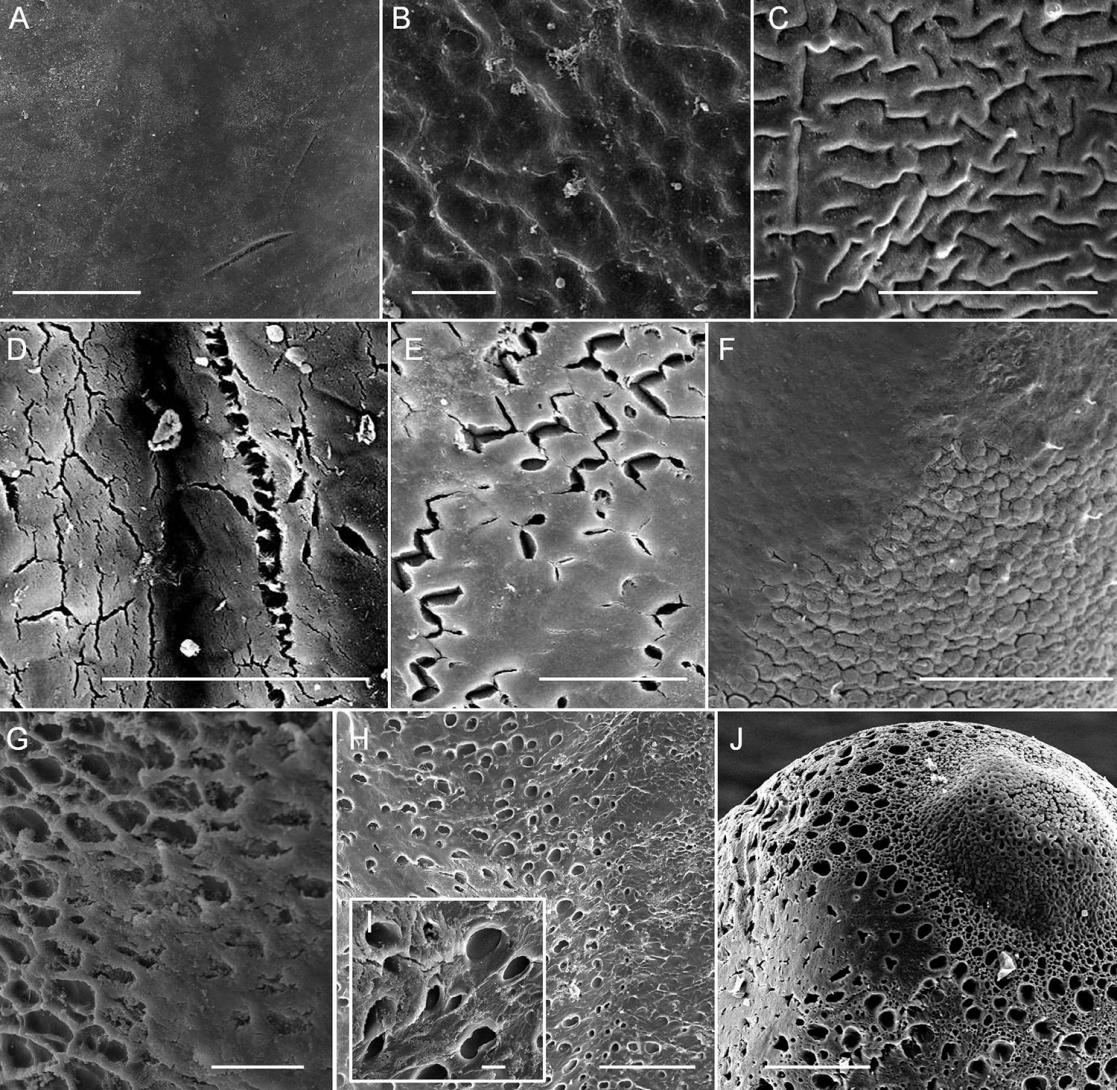
Morphological variation of the cuticle that may result from mucus. **A** Smooth cuticle **B–C** Wrinkled cuticle **D–E** Cracked surface of cuticle **F** Areole-like structures on the anterior end of the cuticle **G–J** Indentations on the **G–I** mid-body and **J** anterior end of the cuticle surface. Scale bars 100 µm (**A**), 10 µm (**B**), 50 µm (**C–E**), 100 µm (**F**), 20 µm (**G**), 100 µm (**H**), 10 µm (I), and 100 µm (**J**).

**Figure 6.**
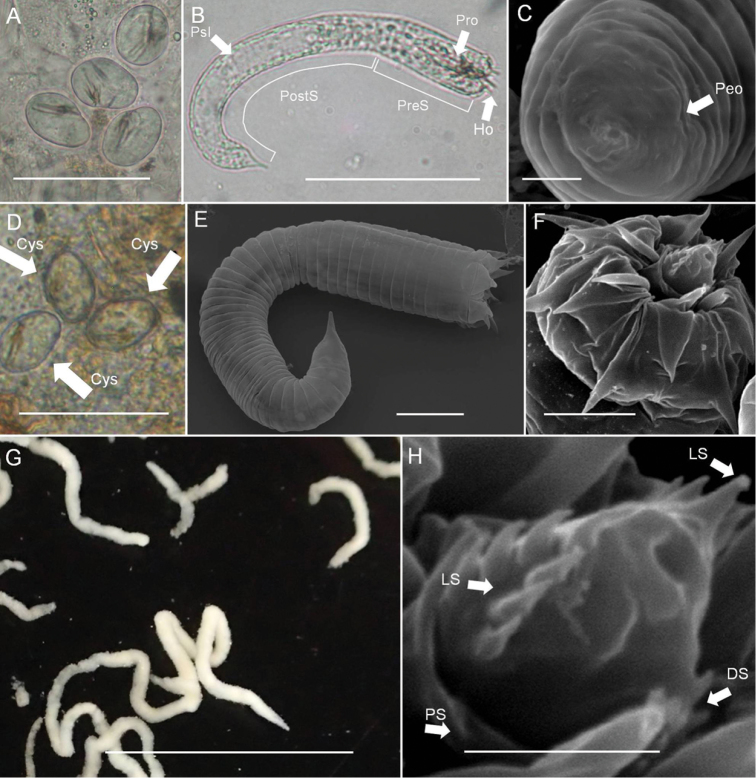
Immature stages of *Acutogordius taiwanensis* sp. n. **A–B** Live **A** cyst-form and **B** worm-form larvae in water **C** Posterior view of a worm-form larva **D** Cysts in an infected snail; **E** Worm-form larva under SEM **F** Anterior view of a larva showing the hook arrangement **G** Egg strings **H** Close-up of the proboscis. DS, dorsal spines; Ho, hooklet; LS, lateral spines; Peo, pseudointestine exterior opening; PostS, postseptum; PreS, preseptum; Pro, proboscis; PS, proboscis sheath; PsI, pseudointestine gland. Scale bars 50 µm (**A–B**), 2 µm (**C**), 50 µm (**D**), 10 µm (**E**), 5 µm (**F**), 1 cm (**G**), and 2 µm (**H**).

**Figure 7.**
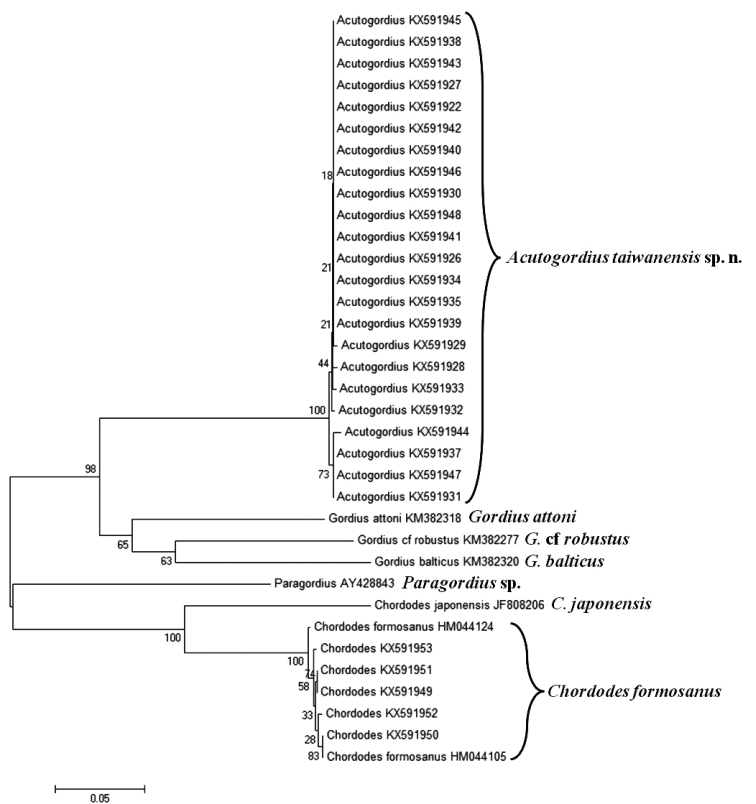
Neighbor-joining tree of *Acutogordius taiwanensis* sp. n. and *Chordodes formosanus* compared to *C. japonensis*, *Gordius attoni*, G. cf robustus, *G. balticus*, and *Paragordius* sp. Numbers at the nodes represent the percentage of 5,000 bootstrap replicates.

**Description** (Figs 1–7). *Male adults* (*n* = 14) (Figs 2, 3). Body length 288.3 ± 90.1 (133–428) mm, width (widest, after dehydration) 623 ± 173 (404–1079) µm. Body light brown, smooth, and slightly mucous covered (liquid on the body surface slightly viscous, light on live worms usually refracted) before fixed in alcohol, alcohol-preserved specimens significantly flat and hard.

Anterior end columnar and slightly narrowed at tip; anterior tip white (white cap) with a dark-brown collar (Fig. 1A); white spots scattered on brown collar (Fig. 1B, C) in some samples (3/14); under SEM, surface of anterior end wrinkled (Fig. 1F) (4/14), smooth (Fig. 1D) (7/14), or smooth but wrinkled on the tip (Fig. 1E) (3/14); short bristles or holes scattered on some samples; no obvious boundary between the white cap and dark-brown collar.

Cuticle in mid-body smooth, slightly wrinkled, or cracked; short or cone-like bristles (Fig. 3D, E) scattered on some samples (6/14).

Posterior end divided into two tail lobes, each 360.25 ± 53.30 (303.70–489.58) µm in length; lobe tips generally tapered, wrinkled, or covered by moderately flat areoles with short spines amongst areoles; inner side of tail lobes smooth; tiny spines scattered around tip; cone-shaped spines or flat areoles scattered on base behind post-cloacal crescent.

Ventral side of posterior end structured with post-cloacal crescent, cloacal opening, and tiny bristles. One post-cloacal crescent not evident as it was covered by larval cuticle, post-cloacal crescent length (extension along longitudinal axis) 275.48 ± 68.84 (195.78–417.03) µm, width (widest) 44.81 ± 16.21 (18.73–83.01) µm, located near base of tail lobes; post-cloacal crescent slightly curved (Fig. 2B, E) (5/13), nearly at right angle (Fig. 2A, D) (5/13, including two samples reared for laying eggs), or semicircular (which were more slender than the curved or angled ones) (Fig. 2C, F) (3/13). Two ends of post-cloacal crescent extending over (Fig. 2 A, C, D, F) (11/13) or anterior to (Fig. 2B, E) (2/13) starting point of tail lobe bifurcation. Cloacal opening circular or slightly oval-shaped, 26.61 ± 7.86 (14.63–43.23) µm in diameter, 55.50 ± 19.71 (32.55–89.90) µm away from anterior margin of post-cloacal crescent, surrounding depressed area in four samples, no circumcloacal spine. Cloacal openings of four specimens not visible as they were covered by the larval cuticle or by mold. Tiny bristles scattered over ventral side of posterior end except in two samples covered by larval skin or mold; tiny bristles scattered over ventral posterior end and concentrated on tail lobes (Fig. 3B) (3/13), anterior post-cloacal crescent (Fig. 3A) (1/13), or randomly scattered on the cuticle (Fig. 3D, E) (9/13).

*Female adults* (*n* = 10) (Fig. 4). Body length 271.80 ± 99.14 (73–432) mm, width (widest, after dehydration) 896 ± 171 (578–1120) μm, light brown, slightly mucous covered (liquid on the body surface slightly viscous, light on live worms usually refracted) before fixed in alcohol. Alcohol-preserved specimens flat in egg-laying samples. Anterior end (Fig. 4A) columnar and slightly narrowed at tip; white cap and dark-brown collar present. Under SEM, surface of anterior end smooth or wrinkled; one sample had hole-like structures (Fig. 5J); small spines scattered on surface of three samples; no obvious boundary between the white cap and dark-brown collar. Cuticle in mid-body smooth, wrinkled, or crack-like; most with small spines scattered on cuticle (7/10). Posterior end (Fig. 4B) rounded, smooth, without spines or bristles. Cloacal opening on terminal end circular, 24.70 ± 5.88 (16.80–30.62) μm in diameter, no circum-cloacal spine.

*Eggs* (Fig. 6G). Egg string (Fig. 6G) length 12.04 ± 3.91 (4.94–19.13) mm, width 0.61 ± 0.11 (0.343–0.708) mm (*n* = 11), white or light yellow in color, deposited as short pieces not adhering to substrate. Eggs (12 days after being laid, nearly hatching) oval-shaped, length 31.93 ± 3.08 (28.79–34.67) µm, width 25.69 ± 1.25 (24.04–27.71) µm (*n* = 6).

*Larvae* (Fig. 6A–C, E–F, H). Newly hatched larvae near eggs presented as “worm-form” (Fig. 6B, E) or “cyst-form” (Fig. 6A). Both found among crushed egg strings. Under light microscopy, worm form (*n* = 13) larvae pre-septum length 31.25 ± 2.83 (24.66–34.14) μm, width 13.18 ± 0.44 (12.30–14.13) μm; post-septum length 80.75 ± 3.87 (77.16–89.13) μm, width 11.17 ± 0.70 (9.76–2.60) μm. Proboscis (same as stylet in our previous description in Chiu et al. (2011)) length 11.77 ± 0.87 (10.14–12.46) μm, width 3.29 ± 0.39 (2.79–4.02) μm; pseudo-intestines unequally subdivided, oval with length 48.22 ± 2.86 (44.69–54.32) μm, width 7.99 ± 0.87 (6.57–9.17) μm. Cyst form (*n* = 15) larvae post-septum folded into an oval shape, length 25.64 ± 1.66 (22.34–27.88) μm, width 17.41 ± 1.40 (14.91–19.38) μm; proboscis the only obvious structure, length 11.19 ± 1.25 (8.22–13.23) μm, width 2.60 ± 0.63 (1.38–3.21) μm.

Under SEM (worm-form larvae), larvae superficially annulated with 13 segments on pre-septum and 35 on post-septum, ectodermal septum not distinguishable (Fig. 6E). Hooks arranged in three rings on anterior pre-septum: outer ring containing seven hooks, including ventral double hooks close to each other; middle and inner rings containing six hooks, and six inner spines, located between each outer hook (Fig. 6F). Proboscis inside the pre-septum covered by sheath, ornamented with two sets of spines: seven larger spines arranged laterally in two lines, except the largest terminal spine; seven smaller spines on dorsal side, no spines on ventral proboscis (Fig. 6H). One single posterior spine located on end of post-septum (Fig. 6E); exterior openings of pseudo-intestine may be present, but not clear (Fig. 6C).

*Field-collected cysts* (Fig. 6D) Three cysts inside a snail length 23.59–24.35 μm, width 15.33–16.45 μm; proboscis length 11.42–11.91 μm, width 1.67–2.047 μm. Shape of cysts similar to cyst-form larvae, no cyst wall found, likely ruined during sample preparation.

#### Phylogeny.

Except for one female with insufficient DNA for sequencing, the 23 *Acutogordius* COI sequences (GenBank numbers KX591922, KX591926–KX591935, KX591937–KX591948) contained eight haplotypes with 442 invariable sites, six singletons, and two parsimoniously informative sites. The genetic distance among them was 0.0025 with a range of 0.0000–0.0112. The phylogenetic tree had a polytomic topology in which some clades were not highly supported because of low bootstrap values and short genetic distances (Fig. 7). The genetic distance between the COI sequences of these 23 *Acutogordius* individuals and that of *G. balticus* was 0.27948 compared to 0.25455 and 0.27439 for *G. attoni* and G. cf. robustus, respectively.

#### Comments.

The 23 *Acutogordius* samples from orthopteran hosts were determined to be from a single species based on their low genetic distances, which was similar to the intraspecific pairwise distances found within G. cf. robustus (0.64–2.63%) (Hanelt et al. 2015) and *C. formosanus* (0–1.92%) (Chiu et al. 2011) and lower than the interspecific pairwise distances among species of the genera *Gordius* (8.0–24.3%) (Hanelt et al. 2015) and *Chordodes* (16.84%) (Chiu et al. 2011).

All three morphological types of post-cloacal crescents identified in *A. protectus* were apparent in the *Acutogordius taiwanensis* sp. n. samples. Nevertheless, post-cloacal crescents significantly extending onto the tail lobes were only described in *Acutogordius taiwanensis* sp. n. and previously in *A. acuminatus* de Miralles and de Villalobos 1998, *A. feae* (Camerano, 1897), *A. obesus* (Camerano, 1895), and *A. sulawensis* Schmidt-Rhaesa and Geraci, 2006. High intraspecific variation in the post-cloacal crescent makes this structure unsuitable as a diagnostic characteristic at the species level, despite that it is the most obvious structure that can be examined by stereomicroscope.

Short bristles on the mid-body were a newly described character, which were first found in *A. finni* (Schmidt-Rhaesa and Schwarz 2016). This character is not likely to be examined by stereomicroscope, but in *Acutogordius taiwanensis* sp. n., the short bristles were still not consistently present in all individuals examined under SEM. One of the possible reasons is that the bristles were covered by mucus on the cuticle surface. The surface of *Acutogordius* has been generally described as totally smooth (de Miralles and de Villalobos 1998, Schmidt-Rhaesa et al. 2006). However, various structures were found on the surface of *Acutogordius taiwanensis* sp. n., including wrinkled, cracked, or indented structures. A similar structure (fine grooves as described in Schmidt-Rhaesa and Schwarz (2016) have been found in *A. finni* (Figs 4D, E in Schmidt-Rhaesa and Schwarz (2016)), and some of the bristles look like “sticks” on the cuticle surface. In addition, the areole-like structure on the anterior end of one female, also suggested the possibility that the moderately flat areoles covering male tail tips were caused by mucus. Thus, although the moderately flat areoles and short bristles were applied as the main diagnostic characters for *Acutogordius taiwanensis* sp. n. and *A. protectus*, more information may be necessary to confidently distinguish between the two species.

### Chordodes formosanus

Taxon classificationAnimaliaNematomorphaGordiida

Chiu, 2011

#### Material examined.

Taipei Zoo (24°59′44.70′′N, 121°34′49.49′′E), Taipei City, Taiwan (three males from an *Acromantis japonica* individual); Wufengqi Waterfalls (24°49′55.62′′N, 121°44′50.10′′E), Jiaushi Township, Yilan County, Taiwan (two males from two Tettigoniidae species). For specimen details, see Table 1.

#### Hosts.

*Acromantis japonica* Westwood, 1889 (Mantodea: Mantidae). *Leptoteratura* sp., *Holochlora japonica* Brunner von Wattenwyl, 1878 (Orthoptera: Tettigoniidae). For host details, see Table 2.

#### Redescription

**Figure 8.**
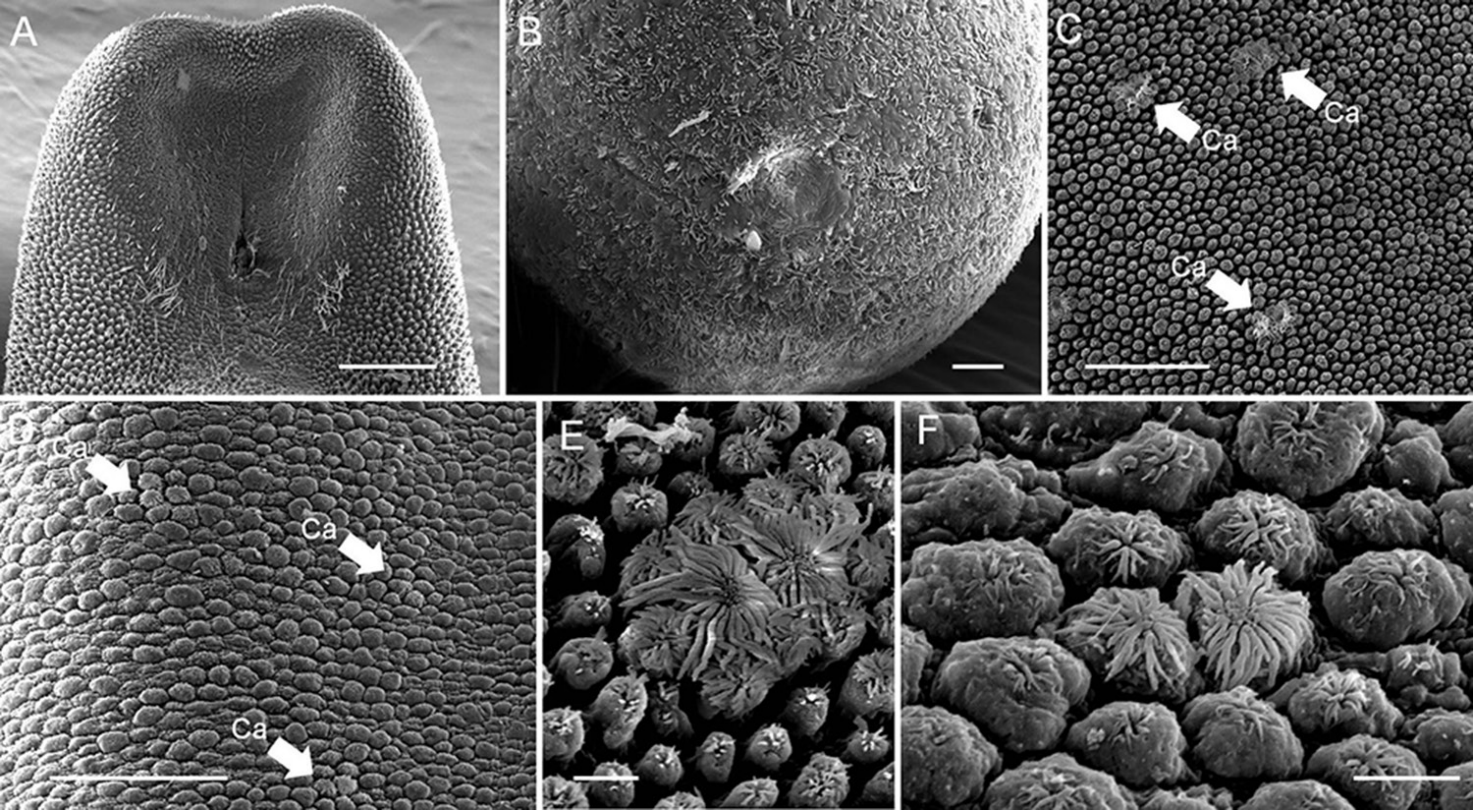
Male adult *Chordodes formosanus* from novel hosts. **A** Posterior end **B** Anterior end **C–D** Variable crowned areole morphologies from different individuals **E** Close view of (**C**) with typical *C. formosanus* crowned areoles; **F** Close view of (**D**) with smaller crowned areoles. Ca, crowned areole. Scale bars 100 µm (**A**), 10 µm (**B**), 100 µm (**C–D**), and 10 µm (**E–F**).

(Fig. 8). *Male adult* (*n* = 5). Body length 109 ± 64 (43–204) mm, width (widest, after dehydration) 0.56 ± 0.29 (0.32–0.88) mm, dark-brown, rough, and flat with dorsal and ventral grooves in alcohol-preserved specimens.

Except for one sample with the broken posterior end, which was not described, the posterior end of the other 4 samples (Fig. 8A) not lobed, ornamented areoles on margin with short spines between them. Oval cloacal opening subterminal, 61.14 ± 27.61 (44.41–93.00) μm long and 31.23 ± 11.42 (22.00–44.00) μm wide, circum-cloacal spines present. A pair of oval regions free with areoles posterior to cloacal opening with scattered bristles over it. Paired oval bristlefields 171.94 ± 48.32 (127.84–223.59) μm long and 55.94 ± 10.08 (46.34–66.43) μm wide, not found in one sample, located on lateral side of cloacal opening between areas adjacent to flat areoles and normal areoles. Anterior end (Fig. 8B) tapered, with white tip (white cap) under stereomicroscopy. Under SEM, anterior tip smooth or wrinkled, covered with abundant small spines, and scattered, thick bristles; mouth open on terminal end of anterior extremity.

Mid-body covered with areoles with some ornamentation on surface. Areoles characterized into five types (simple, tubercle, thorn, circumcluster, and crowned areoles). Simple areoles, most abundant, covering entire cuticle of mid-body, 9.70 ± 1.84 (8.18–12.51) μm in diameter, circular, surface smooth or uneven. Tubercle areoles and thorn areoles scattered among simple areoles, similar in shape, but with a tubercle (ca. 3.96–8.09 μm long) or a solid thorn (ca. 7.35–16.92 μm long), respectively, on the latter or on top of thorn areoles; thorn areoles less abundant than tubercle areoles and not found in one sample. Crowned areoles (Fig. 8C, E) (each 14.90 ± 3.40 (9.72–21.81) μm in diameter) surrounded by 7–12 circumcluster areoles with a central tubercle in between; each areole with a flat top and medium filaments (13.11 ± 4.96 (7.41–27.62) μm) originating from the apical center to edges; few long filaments (55.43–179.70 μm) found in one sample. In one small individual from *Acromantis* mantid, crowned areoles (Fig. 8D, F) smaller than usual (10.84 ± 0.84 (9.60–11.83) μm) and apical filaments (6.56 ± 1.11 (5.03–8.36) μm), with almost same-sized circumcluster areoles.

#### Phylogeny.

The genetic distances among all horsehair worms from *Acromantis japonica* (GenBank nos: KX591949- KX591951), *Leptoteratura* sp. (GenBank no.: KX591952), *Holochlora japonica* (GenBank no.: KX591953), and *Hierodula* mantid (sequences from Chiu et al. (2011)) COI sequences ranged from 0.000 to 0.010. The phylogenetic tree (Fig. 7) revealed a polytomic topology, whereas the five horsehair worm sequences from the *Acromantis* mantid and Tettigoniidae hosts were randomly inserted into this clade.

#### Comments.

The five male horsehair worms were all determined to be *C. formosanus* because of the low genetic distances between their COI sequences and those of *C. formosanus* individuals as described in Chiu et al. (2011). Their morphology was also similar to that of the species described in Chiu et al. (2011), which consisted of five areole types in male adults with slight differences found in the bristlefield and crowned areoles.

The size of the bristlefields was smaller in individuals in the present study than in those described in Chiu et al. (2011) (70–77 μm wide and 145–243 μm long, respectively) and were not found at all in one extremely small individual. Although the difference in the bristlefields in the male *Chordodes* species has not been used to distinguish the species, this character has been used to distinguish *Gordionus kii* from *G. chinensis* (Schmidt-Rhaesa and Sato 2009).

Another abnormal morphological feature was the similar-sized paired crowned areoles and their surrounding circumcluster areoles. These “abnormal crowned areoles” were only found in one extremely small individual, but not in the other horsehair worms, including the two large ones that emerged from the same host individual. Because the molecular data suggested that this individual was conspecific with *C. formosanus*, we believe the abnormal crowned areoles may have been caused by incomplete development during synchronized maturation (see Discussion for details).

## Discussion

In this article, a new species, *Acutogordius taiwanensis* sp. n. and its immature stages were described, and 11 species of orthopteran insect hosts of this new species were identified. In addition, three novel hosts of *C. formosanus*, *Acromantis japonica*, *Leptoteratura* sp., and *Holochlora japonica*, were identified.

### *Acutogordius taiwanensis* sp. n.

*Intraspecific variation.* Finding stable diagnostic characters is a crucial step in distinguishing horsehair worm species (Schmidt-Rhaesa and Geraci 2006, Hanelt et al. 2015). This process includes two main challenges: 1) finding a stable diagnostic character and 2) setting the boundary between intra- and interspecific variation. The postcloacal crescent was the main diagnostic character distinguishing the *Acutogordius* and *Gordius* species (Schmidt-Rhaesa 2001). This structure, despite its function being unclear, is easily examined under both an SEM and a stereomicroscope. However, its morphology may be more unstable than previously understood. The potential polymorphism has been documented in *A. protectus*, which has primarily been characterized by tail areoles, instead of the postcloacal crescent (Schmidt-Rhaesa and Geraci 2006). In *Acutogordius taiwanensis* sp. n., distinct intraspecific variation in the postcloacal crescent was further confirmed. Likewise, the small ornamentations on the body cuticle currently described as a diagnostic character in *Acutogordius taiwanensis* sp. n. and *A. finni* (Schmidt-Rhaesa and Schwarz 2016) may also appear in other *Acutogordius* species, especially those that were described without SEM examination before the descriptions of de Miralles and de Villalobos (1998) and Schmidt-Rhaesa et al. (2006).

Using SEM to describe nematormorph species has become standard practice (Schmidt-Rhaesa 2001). However, mucus, which may be present in *Acutogordius* (as well as *Gordius*), is likely to obstruct various morphological features under SEM. This is the first report suggesting that horsehair worms may secrete mucus on its body surface, and the function of this mucus is unknown. As an aquatic animal that parasitizes terrestrial hosts, the horsehair worm is known to avoid emerging on land where it could become dehydrated by manipulating its hosts (Thomas et al. 2002). The mucus secretion could be an additional strategy for retaining moisture caused by the high risk of being out of the water.

Because of high intraspecific variation, the conspecific status of the 28 examined samples of *Acutogordius taiwanensis* sp. n. was primarily based on the comparison of DNA barcodes and secondarily supported by the similarity of hosts and habitat. DNA sequencing, with the application of SEM since 1980 (reviewed in Chandler and Wells 1989), provides a new and standard tool in horsehair worm taxonomy. Although the database of horsehair worm sequences is not yet complete enough to enable determination of a new species, it is useful in judging the conspecific status of a set of samples. The combination of molecular and morphological data has also improved our understanding of intraspecific variation and cryptic species (Chiu et al. 2011, Hanelt et al. 2015). Thus, while here we suggested that *Acutogordius taiwanensis* sp. n. is a newly described species to science, its phylogenetic relationship with nine other *Acutogordius* species (or seven species, see Schmidt-Rhaesa 2002) is still unclear and worth further investigation.

*The immature stages of Acutogordius*. Studies of the immature stages of horsehair worms have received more attention in recent years. Although morphological identification of the immature stage to the genus level is only now roughly possible (Szmygiel et al. 2014), cysts from a wide range of aquatic hosts have recently been used to estimate geographic distributions (Hanelt et al. 2001, Harkins et al. 2016), species composition (Bolek et al. 2013a), and annual reproductive seasons (Chiu et al. 2016). The morphology of the immature stages of *Acutogordius* in the present study was similar to that of the cysts we found in a field survey (Chiu et al. 2016, Fig. 1, type 2 cyst), which suggests the distribution of *Acutogordius*, despite the fact that no adult worms were found during that survey.

Newly hatched larvae that fold their bodies outside of hosts have rarely been described (Dorier 1930, Bolek et al. 2015). This might not be typical, because larvae of numerous gordiid species have never been observed to encyst in air or water, and species folding their larvae have only occasionally been observed in some individuals (Bolek et al. 2015). In the case of *Acutogordius taiwanensis* sp. n., the folded larvae (cyst form) were found among the crushed egg strings. As there were some dead larvae near the egg strings, it seems the eggs had already hatched for a few days before being examined. It is uncertain if the larvae folded outside of the eggs or inside the eggs and then were pushed out. Nevertheless, because all the larvae inside the eggs were found to be unfolded, we believe they folded themselves at the egg strings after hatching. To date, we do not yet know which factors triggered newly hatched larvae to become worm-shaped or cyst-shaped because both were found under similar living conditions. Larval worms are thought to stay on the river bottom and be passively ingested by paratenic hosts (Hanelt et al. 2005). Folding their bodies may provide a benefit by reducing the water flow or by making them easy to ingest because the postseptum in *Acutogordius*, as well as in *Gordius*, is significantly longer than in other genera (Hanelt and Janovy 2002, Szmygiel et al. 2014).

*Acutogordius taiwanensis* sp. n. hosts. Prior to this study, the only reported host of *Acutogordius* was *Acanthodis* sp. (reviewed in Schmidt-Rhaesa 2013). In the case of *Acutogordius taiwanensis* sp. n., it was found to emerge from several families of Orthoptera with different foraging behaviors. Most of the hosts are obligate or facultative predators and are frequently found to prey on small insects in the field (e.g., *Eugryllacris* sp., *Neanias magnus*, *Hexacentrus japonicus*, and *H. unicolor*), but some of them are primarily herbivorous in Taiwan (e.g., *Deflorita apicalis*, *Elimaea* sp., *Isopsera* sp., and *Phaulula* sp.). Adult horsehair worms have emerged from non-carnivorous hosts, herbivores (*Barbitistes serricauda*, *Leptophyes punctatissima*), or detritivores (*Cambala annulata*) (Schmidt-Rhaesa et al. 2005, 2009). These horsehair worms challenge our current knowledge because these worms typically invade their definitive hosts through paratenic hosts, which require that a carnivorous host ingests a paratenic host carrying the horsehair worm’s cyst (Hanelt et al. 2005). A possible pathway for parasitizing these non-carnivorous hosts is via consumption of larvae/cysts in the water or on vegetation (Schmidt-Rhaesa et al. 2005, 2009). Horsehair worm cysts have known to maintain partial infectiousness after the paratenic host dies and after they are dry for 30 days (Bolek et al. 2013b). This makes them capable of being accidentally ingested by herbivorous or detritivorous, if the dead paratenic host is in the water or on the vegetation. In addition, it is also possible that herbivorous hosts facultatively prey on weak or newly emerged paratenic hosts. Regardless of the pathway the horsehair worm cysts follow, the infection rates of non-carnivorous hosts are theoretically lower than that of predators, and this supports our observations in the field in Taiwan.

### Chordodes formosanus

*Novel hosts*. The *Acromantis* mantid and *Leptoteratura* sp. are general predators that can easily ingest the cysts of *C. formosanus* in paratenic hosts, but *C. formosanus* was previously thought to develop specifically inside *Hierodula* mantids (Chiu et al. 2011). The novel hosts suggest flexibility in *C. formosanus* host use. Such a phenomenon may also occur in *C. japonensis*, which primarily parasitizes *Tenodera* mantids (Inoue 1952, 1955; Chiu et al. 2011), but has also been found in long-horn grasshoppers, *Hexacentrus japonicus japonicus* (Inoue 1955).

In the present study, we recorded long-horn grasshoppers and an *Acromantis* mantid as hosts of *C. formosanus*. However, we do not think these novel hosts are the general ones used by *C. formosanus*. Horsehair worm hosts have been recorded for most of horsehair worm genera (Schmidt-Rhaesa 2013), but knowledge of host preference, the extent to which a particular host taxon is used by a parasite (Lymbery 1989), is generally lacking. We believe that *Acromantis* mantids and tettigoniids, compared to *Hierodula* mantids, are rarely parasitized by *C. formosanus* because of the seasonal infection rates of aquatic paratenic hosts in Taiwan. Our previous survey of horsehair worm infections in aquatic paratenic hosts suggested a single infection peak after the adult *C. formosanus* emerge from *Hierodula formosana* individuals. Such an infection peak did not appear, at least not significantly, after the spring when adult worms emerged from *Acromantis* mantids and tettigoniids (Chiu et al. 2016, Fig. 1). Thus, the contribution of *Acromantis* mantids and tettigoniids to the population of *C. formosanus* may be less than that of *Hierodula* mantids. It is not clear if development in novel hosts is ecologically significant or accidental, but flexibility in host use may improve our understanding of the physiological mechanisms triggering cyst metamorphosis.

*Abnormal morphology of the smallest individual.* Horsehair worm length is strongly correlated to the host’s size and to the number of individuals in a single host (Hanelt 2009). However, the relationship between horsehair worm size and its morphology has not been evaluated. The only individual bearing “abnormal crowned areoles” and lacking bristlefields was half the length (58 mm) of the other two (125 and 115 mm) individuals from the same host individual. One hypothesis explaining the abnormal morphology, even though we have no direct evidence, may be related to incomplete development. Thus, the morphological similarity of the abnormal crowned areole and the simple areole might suggest the crowned areole is differentiated from the simple areole. The similarity of the (well-developed) crowned areole and the simple areole were also suggested by the ultrastructure of *Chordodes nobilii* examined by transmission electron microscopy (Schmidt-Rhaesa and Gerke 2006).

The possible reason causing incomplete development may be resource competition and synchronized maturation. Horsehair worms inside a host individual may compete for resources to increase their fecundity, or they may ensure their survival by synchronizing maturation before the host performs the suicide behavior. These actions would subsequently cause the horsehair worm, which may enter the host later than its neighbors, to mature without completing its development. Thus, we suggest that the abnormal crowned areoles may be a result of incomplete development rather than small size. This hypothesis could also be supported by another extremely small *C. formosanus* (43 mm in length) with crowned areoles that were more likely to be normal in this study. This small *C. formosanus* singly developed in a host individual. Without the influence from neighbor horsehair worms, its small size might have been the result of the small host (9.63 mm in length), instead of incomplete development.

## Supplementary Material

XML Treatment for Acutogordius taiwanensis

XML Treatment for Chordodes formosanus
